# Consumer Choices in the Pasta Market: The Importance of Fiber in Consumer Decisions

**DOI:** 10.3390/nu13092931

**Published:** 2021-08-24

**Authors:** Marta Sajdakowska, Jerzy Gębski, Marzena Jeżewska-Zychowicz, Maria Jeznach, Małgorzata Kosicka-Gębska

**Affiliations:** Department of Food Market and Consumer Research, Institute of Human Nutrition Sciences, Warsaw University of Life Sciences (WULS-SGGW), 159C Nowoursynowska Street, 02-776 Warsaw, Poland; jerzy_gebski@sggw.edu.pl (J.G.); marzena_jezewska_zychowicz@sggw.edu.pl (M.J.-Z.); maria_jeznach@sggw.edu.pl (M.J.); malgorzata_kosicka_gebska@sggw.edu.pl (M.K.-G.)

**Keywords:** consumer choices, fiber, pasta, pasta with added fiber

## Abstract

The aim of the current study was two-fold: (1) to identify consumer segments based on pasta selection motives and (2) to examine the differences between the identified segments in terms of perception of pasta and pasta with added fiber and information on the food label. The data were collected using a CAPI (computer-assisted personal interview) survey on a sample of 1013 consumers. The k-means clustering method was used to identify four clusters of consumers, namely, quality-oriented, sensory-oriented, convenience-oriented, and neutral consumers. The quality-oriented group was the group that expressed the most positive opinions about the pasta and about the addition of fiber to pasta. Moreover, they appreciated the information placed on the pasta label the most. Consumers in the sensory-oriented segment were the least likely to agree that the addition of fiber to pasta deteriorated its taste and to agree that it looked worse compared to pasta without fiber. These findings are of significance for those involved in the public nutrition sector as well as for those responsible for preparing well-targeted marketing messages. The conclusions may constitute invaluable insights for those devising educational initiatives and campaigns.

## 1. Introduction

Today’s customers have limited time to prepare meals and, thus, convenience food is sought after [1]. At the same time, a growing interest in a healthy diet has been observed [2,3]. Consequently, consumers’ perceptions and purchasing behaviors are influenced by their health awareness [4].

Dietary fiber is a promising food ingredient with health benefits [5]. Moreover, dietary fiber is involved in disease prevention and enhances the health of consumers [6]. Epidemiological and short-term interventional studies emphasize the association between a higher fiber intake and improvements in the lipid profile as well as fasting and postprandial glycemic control. Some fractions of fiber are more effective, e.g., for the management of diabetes, obesity, dyslipidemia, and hypertension [7]. However, the current intake of fiber is still far below the recommended level in most nations worldwide [8]. Although the consumption of wholegrain foods has been encouraged due to the association between whole grains and health benefits, changes in the technological parameters and sensory attributes may limit the consumption of these products [9].

Consumers’ preferred staple foods, such as bread and pasta, as base products for modification [10]. Fresh noodles enriched with fiber-rich fractions contribute to food convenience due to improved nutritional quality, reduced cooking time, and acceptable cooking quality [11]. A product with a changed composition is in greater demand if there is an acceptance of the product as a carrier of the added ingredient [12]. Therefore, the motives for selecting pasta as a food product that is convenient to use may be crucial in assessing the acceptance of a product with a modified composition, i.e., pasta with added fiber. Adding various sources of fiber to pasta can result in lowering calorie intake by manipulating starch degradation [6,13]. Moreover, product modifications can worsen its physical, chemical, and sensory properties and these properties are vital for consumers’ acceptance [14,15]. Consequently, in recent years, dietary fiber has been used in improving pasta [16,17,18,19,20,21].

The food industry aimed to enhance the overall nutritional balance of carbohydrate-rich foods by raising their dietary fiber content at the cost of readily digestible carbohydrates. Moreover, the food industry can use the physicochemical properties of fiber to enhance some properties of their products, such as viscosity, texture, sensory characteristics, and shelf-life [6]. Furthermore, fiber-enriched pasta could be produced by increasing the content of dietary fiber by several percent in a regular semolina-based pasta formulation, leading to acceptable products with matching characteristics of texture and color compared to commercial products [22]. Thus, both consumers and the food industry may benefit from enriching cereal products with dietary fiber components [23,24].

Thus, the aim of the current study was two-fold: (1) to identify consumer segments based on pasta selection motives and (2) to examine differences between the identified segments in terms of the perception of pasta and pasta with added fiber, and information on the food label.

## 2. Materials and Methods

### 2.1. Data Collection Process

The sample used in the study (N = 1013) was obtained through a cross-sectional quantitative survey, being part of a Bioproduct project. The following paper discusses selected findings from a larger study [25,26]. The sample was selected using the following criteria: the representativeness of the population of Poland according to voivodship and the quota character by gender, education, and place of residence. All subjects of the study were 21+. Only those respondents who met other recruitment criteria, i.e., made their own or cooperative food purchase, participated in the study. A professional market research agency was used to conduct interviews with respondents. The interviews were performed on a face-to-face basis at respondents’ homes. Moreover, the ESOMAR (European Society for Opinion and Marketing Research) code of conduct was respected and the CAPI (computer-assisted personal interview) technique was employed.

### 2.2. Description of Questionnaire

The questionnaire in the study comprised a few main sections and discussed issues, such as: (A) the importance of pasta selection motives (“How important are the following factors for you when purchasing pasta?”, where 1 = not important at all and 5 = very important) (items are presented in Table 1) as well as (B) the lifestyle self-assessment (“How do you assess yourself in terms of your lifestyle?”, where 1 = I totally disagree and 5 = I totally agree) (statements are presented in Table 4). In order to evaluate (C) the consumers’ opinion regarding pasta, and the importance of food information, including the significance of information on the pasta label, the following questions were asked: “To what extent do you agree with the following statements on pasta?”, “To what extent do you agree with the following statements?”, where 1 = I totally disagree and 5 = I totally agree; and “How important is the following information on the pasta label for you?”, where 1 = not important and 5 = very important (statements are presented in Tables 5 and 6). Finally, in order to identify opinions on cereal products with added fiber and opinions on pasta with added fiber compared to the same pasta without added fiber, the questions were worded as follows: “To what extent do you agree with the following statements about grain products with added fiber?” and “Please compare pasta with added fiber to the same pasta without fiber”, where 1 = I totally disagree and 5 = I totally agree (items are presented in Tables 7 and 8).

### 2.3. Statistical Analysis

Analysis of the statements’ reliability in a question regarding the motives for pasta choice was performed using the Cronbach coefficient alpha. The value of the Cronbach coefficient alpha = 0.908 confirmed the right choice of questions for factor analysis (FA). The factors obtained via the FA explained 59.6% of the total variation. Particular factors were qualified based on the minimum value of factor loadings determined at 0.5, with the factor adequate for the requirements of factor analysis as studied by the Kaiser-Mayer-Olkin measure (KMO). The KMO value, indicating collective correlation of variables, was 0.922, which clearly validated the rationale behind employing the variable reduction method. Table 1 presents the identified factors that were used for cluster analysis.

Consumers were divided into segments in a two-stage process. The first stage consisted in performing a cluster analysis using hierarchical methods. The second stage included a cluster analysis based on non-hierarchical method k-means with initial cluster seeds, which emerged using the hierarchical method. Four well-separated clusters were achieved (Table 2).

Moreover, the mean values of opinions between pairs of clusters were compared by means of a post-hoc test (Waller-Duncan k-ratio t-test). Taking the motives for choosing pasta into account, consumers in segment No. 1 valued sensory motives and availability most. In the case of segment No. 2, the most important were the sensory motives and availability, and the least importance compared to other segments was attached to the convenience and familiarity. For the consumers in segment No. 3, the most important were the convenience and familiarity and the marketing motives were the least important. In the case of segment No. 4, the marketing motives were rated lower than in the case of segment No. 1, whereas the least importance was attached to the sensory motives and availability.

The statistical analysis was carried out using the SAS 9.4 statistical package (SAS Institute, Cary, NC, USA).

## 3. Results

### 3.1. Description of the Sample and Clusters

As previously indicated, the non-hierarchical k-means clustering method led to the identification of four clusters: Quality-oriented (cluster 1), sensory-oriented (cluster 2), convenience-oriented (cluster 3), and neutral (cluster 4) clusters. Socio-demographic variables, such as gender, age, education, size of the place of residence, and subjective assessment of the financial situation, were used to profile the clusters (Table 3). However, variables, such as gender and age, did not significantly influence the profile of the clusters. The independence *χ*^2^ test was used to assess the diversity of profile features between clusters. The characteristics of the study sample are shown in Table 3.

Segment No. 1 (quality-oriented; N = 245; 24.2%) comprised more than 1/3 people with secondary education (35.51%), and every 5th respondent surveyed had higher education (20.41%), which is slightly less than in segment No. 2. In total, 40.0% of the respondents in segment No. 1 declared rural areas as their place of residence, and 15.1% of people also resided in a city of more than 500,000 inhabitants, which is the highest percentage compared to the other segments. Taking income into consideration indicates that more than half of the surveyed respondents (53.47%) reported that they can afford some but not all of their expenses, and 1/4 reported that (26.53%) income allows them to meet only basic needs.

In segment No. 2 (sensory-oriented; N = 221; 21.8%), more than 1/3 of the surveyed (36.2%) were respondents with secondary education and more than 1/4 of the surveyed (26.2%) were respondents with higher education. It should be noted that this represented the largest percentage compared to the other segments. Taking into account the place of residence indicated that, as in the case of segment No. 4 (described later), almost 4/5 of the respondents lived in rural areas (38.5%) and 1/4 (24.0%) lived in a town of less than 50,000 inhabitants. Only every 10th respondent (10.4%) declared that they live in a city with a population between 101,000 and 300,000, which is the lowest percentage compared to the other segments. Taking into account the subjective assessment of the financial situation, it was indicated that half of the surveyed individuals (52.0%) declared that they could afford some but not all expenses, and 18.6% stated that their income allowed them to meet only basic needs, while a similar percentage (19.0%) of the surveyed individuals claimed that they could afford everything, and in this category of assessment, this group of respondents was the most numerous in comparison with the other segments.

In segment No. 3 (convenience-oriented; *N* = 216; 21.3%), more than 2/5 of the respondents (42.59%) declared secondary education. Consideration of place of residence indicated that more than one-third (36.57%) were rural residents and almost one-third (29.17%) lived in a city of 101,000 to 300,000 residents. This segment was dominated by people assessing their income as allowing them to meet some but not all of their expenses (64.35%).

In segment No. 4 (neutral; *N* = 331; 32.7%), individuals with secondary education and vocational education comprised approximately 70% of respondents in this segment (35.4% and 33.5%, respectively). Almost 40% of the respondents were residents of rural areas (38.4%), every 5th respondent declared that they live in a town of less than 50,000 residents (21.5%), and a much smaller percentage of respondents declared a city of 101,000 to 300,000 residents as their place of residence (18.1%). The subjective assessment of the financial situation indicated that almost half of the individuals surveyed (47.4%) said they could afford some but not all of their expenses, and more than a third (34.1%) said their family income allowed them to meet only basic needs.

To sum up, the socio-demographic characteristics indicated that in the quality-oriented and sensory-oriented segments, there were more individuals with higher (the sum of bachelor’s and higher) education than in the other segments, and in the convenience-oriented segment, there were more individuals with secondary education. One-third of the convenience-oriented segment were inhabitants of cities of 100,000–300,000, whereas in the sensory-oriented and neutral segments, the majority of respondents lived in villages and towns of up to 50,000 inhabitants (the sum of villages and towns of up to 50,000 inhabitants—about 60%). Consumers in the quality-oriented segment and the sensory-oriented segment rated their financial situation as the best one.

Table 4 shows the self-assessment of lifestyle in the study group. Consumers in the quality-oriented segment perceived themselves mainly as caring for their own health, paying great attention to the naturalness of food, physically active, and with high ecological awareness to a greater extent compared to the other segments. The sensory-oriented segment reported high levels of agreement with most of the lifestyle statements; however, statements describing their lifestyle as physically active and with high ecological awareness reported lower levels of agreement compared to both quality-oriented and convenience-oriented consumers.

The convenience-oriented segment, like the sensory-oriented segment, achieved high levels of agreement with most of the statements describing lifestyles, while in the case of the statements about being family oriented and involved in professional work, the level of agreement did not differ from the segment that included quality-oriented and sensory-oriented consumers.

Respondents from the neutral segment were least likely to agree with most of the proposed lifestyle statements compared to the other segments.

### 3.2. Opinions on Pasta and Information on the Food Label

In Table 5, respondents’ opinions on pasta are presented. Respondents in the quality-oriented segment largely agreed with most statements related to general opinions about pasta and food label information. Only for the statement “the taste of pasta is more important to me than its health benefits” did respondents indicate equal agreement compared to those in the sensory-oriented and convenience-oriented segments.

With respect to agreement with two statements, i.e., (1) In order to improve the health-promoting benefits, fiber can be added to the pasta and (2) it is important to consume enough pasta, the level of agreement was similar in sensory-oriented and convenience-oriented segments. Additionally, regarding the statement “Information on product packaging is very important to me”, respondents from the sensory-oriented and neutral segments had the same lowest level of agreement compared to the other segments.

Segment No. 3 with convenience-oriented consumers reported a high level of agreement (i.e., not much lower than segment No. 4) with the statement “Information on product packaging is very important to me”. Regarding the statement “I compare labels to choose products with the highest nutritional value”, respondents from this segment indicated the lowest level of agreement compared to the quality-oriented and neutral segments. However, this level did not differ from the level of agreement expressed by respondents from the sensory-oriented segment.

Respondents in the neutral segment had the lowest level of agreement with the statement “In order to improve the health-promoting benefits, fiber can be added to the pasta as compared to all segments”. “The taste of pasta is more important to me than its health-promoting benefits” also had a low level of agreement, but this was similar to the level of agreement expressed by the sensory-oriented segment.

The opinions on the information placed on pasta labels are shown in Table 6. The three most important pieces of information were price, shelf life, and the name of the product. The quality-oriented segment showed the highest ratings for most of the information on the pasta label compared to the other segments.

A comparison of segment No. 2 and segment No. 3 (sensory-oriented, convenience-oriented) indicates that some of the information on the pasta packaging was of similar importance to consumers in these segments in choosing foods, i.e., shelf life, product name, producer.

The quality label, on the other hand, was significantly more important to consumers in the sensory-oriented segment compared to the convenience-oriented segment. For consumers in the convenience-oriented segment, price, weight, cooking time, and information on health effects were significantly more important compared to the sensory-oriented segment.

Compared to all segments, the neutral segment rated the following information lowest: price, shelf life, product name, weight, and producer. Information on health effects was also rated lowest by consumers in the neutral segment, but it was not significantly different from the rating in the sensory-oriented segment.

### 3.3. The Importance of Adding Fiber to Cereal Products

Table 7 presents respondents’ opinions on the importance of enriching cereal products with fiber. They showed strong agreement with the opinions that such products facilitate a healthy lifestyle and can lower the negative consequences of an inadequate diet. For opinions regarding cereal products with added fiber, the quality-oriented segment significantly indicated the highest level of agreement for all statements. On the other hand, the neutral segment significantly indicated the lowest level of agreement with most of the given statements. Only the statement “The addition of fiber to cereal products worsens their taste” was rated significantly lowest by respondents in the sensory-oriented segment compared to all other segments. Respondents from this segment also rated the statement “I can prevent disease by eating such products regularly” significantly lower compared to the convenience-oriented segment but significantly higher than respondents from the neutral segment.

Consumers’ views on the pasta with added fiber and pasta without added fiber are presented in Table 8. It shows that the respondents most frequently indicated the statement that pasta with added fiber is more expensive compared to pasta without the addition of fiber. Moreover, in their opinion, pasta enriched with fiber is healthier and more nutritious as well as less calorific when compared to pasta without added fiber.

With respect to pasta with increased fiber levels, the highest significant levels of agreement were again observed in the quality-oriented segment for most statements. For the statement that pasta with increased fiber is more expensive compared to pasta without added fiber, the highest levels of agreement were obtained in the quality-oriented and convenience-oriented segments. In contrast, neutral respondents significantly indicated the lowest ratings for statements that pasta with increased fiber content is more expensive, healthier, has higher nutrient content, is lower in calories, and is harder to be found in stores compared to pasta without increased fiber.

Regarding better taste and worse appearance of pasta with increased fiber compared to pasta without fiber, respondents from the sensory-oriented segment indicated the lowest significant ratings of agreement. Information regarding the visually attractive packaging of pasta without fiber compared to pasta with increased fiber was equally important to respondents from all separated segments.

## 4. Discussion

The variances in the factors influencing the choice of staple foods [10,27] should be taken into consideration while researching the acceptance of reformulated foods. Consequently, the research was designed to determine consumer groups according to their pasta selection motives.

### 4.1. Motives for Choosing Pasta

The obtained results showed that the information found on product packaging was important to respondents and that they compared the information on the labels of different foods before making a choice. This information was particularly essential to those in the quality-oriented segment compared to the other segments. Literature research confirms that the details on the packaging of a cereal product [28,29,30], including the information on the label, is significant for buyers [10,31]. In relation to pasta, becoming familiar with the information provided on the labels of food products, and perceiving oneself as a person who cares about health contributed to declaring a willingness to consume pasta with the addition of fiber [32]. However, surveys also showed that consumers do not always refer to the information presented on the packaging, e.g., because they are in a hurry, or the information is too detailed. Moreover, some consumer groups, namely athletes, consumers with health conditions, and those who attach great importance to a healthy lifestyle, may find appropriate food labeling useful [33].

At the same time, it should be emphasized that price and expiration date were among the two most important pieces of information indicated on the label. Similarly, as indicated earlier, these pieces of information were paramount for respondents from the quality-oriented segment. They also declared to a greater extent that they buy more expensive pasta, because in their opinion, the price of the product is adequate to its quality. The importance of price in a food choice is also confirmed by other literature studies [34,35,36]. In the case of information indicating the expiration date, the literature shows that consumers place a high value on the expiration date/shelf life and suitability for consumption [33,37]. Besides, freshness [38,39,40] and food safety [41,42] are important for consumers. Furthermore, information on packaging, including best-before dates, can be a kind of confirmation of food safety [43,44,45].

### 4.2. Choosing Pasta with Added Fiber

Regarding the fiber content of pasta, the subjects of the study declared that it was worth increasing fiber levels to increase the health-promoting benefits of the product. Again, quality-oriented consumers agreed the most, while neutral consumers agreed least with the above-mentioned opinion. When it comes to the addition of fiber to cereal products in general, the most important aspects indicated by respondents were the facilitation of a healthy lifestyle and the reduction of the adverse effects of a poor diet. Again, quality-oriented consumers agreed with this statement to the greatest extent, while neutral consumers agreed to the least extent.

It is estimated that consumers will increasingly make a food choice based on health-related motives. This is due to their value system in which health is ranked high [46,47,48]. The positive perception of health among some consumers results from their pro-healthy diet, which is rich in plant-origin foods (fruit and vegetables) [49,50,51,52]. Views on the health concerns resulting from the presence of fiber in the product are supported by the literature [53,54,55]. Adding dietary fiber to the pasta enables the creation of products with enhanced nutritional value [56] to meet market demands for healthier food choices [57]. Added-fiber grain products appear to be a useful tool for whole-grain avoiders to increase cereal fiber intakes, as this group is unlikely to accept whole-grain sensory properties [58].

Our study indicated that pasta with added fiber in consumers’ opinion is more expensive but also healthier compared to pasta without added fiber. The health aspect was mainly emphasized by quality-oriented consumers. On the other hand, both quality-oriented and convenience-oriented consumers paid attention to the higher price. Consumers in the sensory-oriented segment, in relation to cereal products with increased fiber content, were the least likely to agree that the addition of fiber worsened their taste compared to the other segments, and in relation to pasta with increased fiber content, the least likely to agree that it looked worse compared to pasta without fiber. However, with respect to the taste of pasta with added fiber, these consumers were more cautious, as they were least likely to agree that it has a better taste compared to pasta without added fiber. The cited opinions may indicate that sensory-oriented consumers do not quite like the taste of this pasta, while at the same time, they do not mind that it may have a characteristic darker color and lumps/spots that at the same time visually indicate the presence of fiber (and presumably so they have a visual guarantee/confirmation at the same time that the fiber is there).

Studies in the literature indicate that expectations and sensory experiences are involved in the overall assessment of product quality [59]. Moreover, it has been emphasized many times in the literature that taste plays a major role in food choice [60,61], and for cereal products [62,63,64], including pasta, it was also noted that it was an important selection factor [65]. The literature also indicates that spaghetti fortified with fiber had good overall acceptability, and could represent a healthy product with good technological and sensory properties [21]. Some consumers favored the fortified sample over the control one, including pasta, and some of them would pay more for the fortified products [66]. Generally, consumers showed less acceptance of a modified pasta when a product had a more intense darker color, and bitter or more sour taste. Acceptance grew among consumers who tend to purchase unconventional pasta [67].

Consumers in the sensory-oriented segment participating in our study were also the least likely to report paying attention to recipes for pasta dishes on the label, which may indicate that this segment may most likely have their own well-tried recipes and does not need this type of information on the label. In contrast, recipes for pasta dishes were important to consumers in the quality-oriented and convenience-oriented segments.

Literature studies indicate that consumers are looking for recipes for dishes [68]; however, their availability is important, e.g., recipes on websites are of particular interest due to their ease of use [69]. In the future, online databases providing consumers with, among other things, recipes for various types of dishes in order to compose interesting and nutritious meals from the consumer’s perspective may be of interest [70].

### 4.3. Practical Implications, Strengths and Limitations

The practical implications of our findings for practitioners in the cereal industry as well as for policy makers are that their efforts to influence the consumption of those products should include tailoring them to the specific consumers they aim to target. While developing food products, efforts should be aimed at enhancing the health value of food consumers perceive as convenient and easy to use.

The strength of our results lies in a relatively large sample of the Polish population. Nevertheless, the findings have their limitations. The sample comprised consumers solely or jointly contributing to grocery shopping of a household. Furthermore, the data used in the study was prone to bias. The first concerns the self-declared information obtained from the survey that may be inaccurate due to the unnatural circumstances imposed by the questionnaire itself. Furthermore, the real circumstances in where the choice of pasta is made and the use of products themselves rather than using the questionnaire would reflect the environment where a purchasing decision is made. However, due to the size of the sample as well as logistic and economical limitations, research using the real products or labels was impossible.

For the abovementioned reasons, the findings in this study should be used with caution when cultural differences may occur. Despite these flaws, our study provides new insights into the motives behind pasta selection.

## 5. Conclusions

Finding out about consumers’ motivations and demand for cereal products, including pasta, may be beneficial for manufacturers to design new types of food and devise marketing strategies, which will result in developing a practical and revised approach to attract consumers who want to promote their health, well-being, and quality of life.

There are numerous opportunities for further developments on the market of cereal products with added fiber, e.g., relatively positive opinions on the significance of enhancing fiber in the diet, the acceptance of adding fiber to pasta, and consumer awareness of the beneficial properties of fiber for health. However, it is important to keep in mind which consumer group the fiber pasta is targeted at, because the consumers’ belonging to a particular segment influence what factors are taken into account when making purchasing decisions. Moreover, regarding the fiber content of pasta, quality-oriented consumers agreed the most, while neutral consumers agreed least with the opinion indicating that it was worth increasing fiber levels to increase the health-promoting benefits of the product.

The results of the study can provide valuable insights for those involved not only in nutrition education but also directly for producers and processors operating on the food market. Therefore, these findings are of significance for those involved in the public nutrition sector as well as for those responsible for preparing well-targeted marketing messages. The conclusions may constitute invaluable insights for those devising educational initiatives and campaigns.

## Figures and Tables

**Table 1:** Factor analysis (FA) referring to consumers’ use of pasta selection motives; varimax rotated factor loadings percentage of explained variance (*N* = 1013, Poland).

Pasta Selection Motives	Factor 1 The Sensory Motives and Availability	Factor 2 The Marketing Motives	Factor 3 The Convenience and Familiarity
Taste	0.774	.	.
Use-by date/shelf life	0.718	.	.
General appearance	0.701	.	.
Personal or family preference	0.671	.	.
Price	0.626	.	.
Availability	0.606	.	.
Color	0.518		
Quality label	.	0.748	.
Place of purchase	.	0.748	.
Seller’s opinion	.	0.724	.
Nutritional value	.	0.675	.
Manufacturer/brand	.	0.63	.
Shorter cooking time	.	.	0.806
Knowledge of the product	.	.	0.703
Information on the packaging	.	.	0.617
Package size	.	.	0.593
The variance explained/% explained variance	41.8	11.9	5.9

**Table 2:** Characteristics of the identified segments according to the motives of pasta selection; the mean ratings of the segments on the classification variables.

Pasta Selection Motives	Segment 1	Segment 2	Segment 3	Segment 4	*p*-Value
The Sensory Motives and Availability	3.84 ^b^	4.29 ^a^	2.97 ^c^	1.55 ^d^	<0.0001
The Marketing Motives	4.40 ^a^	2.45^c^	1.68 ^d^	3.20 ^b^	<0.0001
The Convenience and Familiarity	4.05 ^b^	1.58 ^d^	4.46 ^a^	2.22 ^c^	<0.0001

Means with the same letter are not significantly different; ANOVA post-hoc Waller-Duncan K-ratio t Test.

**Table 3:** Socio-demographic characteristics of the consumers surveyed (*N* = 1013, Poland).

Variables	Total Sample	Quality-Oriented 1	Sensory-Oriented 2	Convenience-Oriented 3	Neutral 4	*p*-Value
Sex						0.7759
female	53.4	55.5	54.8	52.3	51.7	
male	46.6	44.5	45.2	47.7	48.3	
Age						0.1902
up to 30 years	20.9	19.2	22.1	21.3	21.2	
31–40 years	17.9	21.2	22.6	16.3	13.3	
41–50 years	16.0	15.5	10.9	17.1	19.0	
51–60 years	18.9	18.8	19.5	17.1	19.9	
over 60 years	26.3	25.3	24.9	28.2	26.6	
Education						0.0001
elementary	6.1	6.5	3.2	3.2	9.7	
vocational	29.4	25.7	26.2	27.8	35.4	
secondary	36.5	35.5	36.2	42.6	33.5	
Bachelor’s Engineer	9.5	11.8	8.2	11.1	7.5	
Higher	18.5	20.5	26.2	15.3	13.9	
Place of residence						<0.0001
village	38.4	40.0	38.5	36.5	38.4	
Town below 50,000	16.3	10.2	24.0	7.4	21.5	
Town from 50,000 to 100,000	13.9	9.4	15.4	13.9	16.3	
City from 101,000 to 300,000	18.7	17.5	10.4	29.2	18.1	
City from 301,000 to 500,000	5.8	7.8	3.6	9.3	3.6	
City over 500,000	6.9	15.1	8.1	3.7	2.1	
Opinion on family income						<0.0001
Is not sufficient at all	5.7	4.5	6.8	4.6	6.7	
Enables to meet only basic needs	26.8	26.5	18.6	24.1	34.1	
We can afford some, but not all expenses	53.5	53.5	52.0	64.3	47.4	
We can afford everything	11.1	12.2	19.0	5.1	9.1	
We can afford everything, and in addition we can put some money aside	2.9	3.3	3.6	1.9	2.7	

*χ*^2^ test of independence, *p*-value < 0.05—differences between groups are significant.

**Table 4:** Description of segments based on the self-assessed lifestyle of the surveyed.

Statements	Mean	Quality-Oriented 1	Sensory-Oriented 2	Convenience-Oriented 3	Neutral 4	*p*-Value
Family-oriented	4.05	4.39 ^a^	4.19 ^b^	4.31 ^ab^	3.53 ^c^	<0.0001
Valuing tradition	3.96	4.34 ^a^	3.90 ^b^	4.21 ^a^	3.54 ^c^	<0.0001
Caring for their own health	3.82	4.06 ^a^	3.90 ^b^	3.82 ^b^	3.58 ^c^	<0.0001
Involved in professional work	3.57	3.91 ^a^	3.66 ^b^	3.73 ^ab^	3.14 ^c^	<0.0001
Attaching great importance to the naturalness of food	3.57	4.07 ^a^	3.55 ^b^	3.66 ^b^	3.13 ^c^	<0.0001
Physically active	3.57	3.91 ^a^	3.45 ^c^	3.74 ^b^	3.27 ^d^	<0.0001
Highly concerned about environment	3.37	3.81 ^a^	3.21 ^c^	3.49 ^b^	3.05 ^d^	<0.0001

Means with the same letter are not significantly different; ANOVA post-hoc Waller-Duncan K-ratio t Test.

**Table 5:** Profile of segments according to statements on pasta and information on food labels.

Statements	Mean	Quality-Oriented 1	Sensory-Oriented 2	Convenience-Oriented 3	Neutral 4	*p*-Value
Information on product packaging is very important to me	3.52	3.86 ^a^	3.26 ^c^	3.67 ^b^	3.32 ^c^	<0.0001
I compare information on product labels before I decide which product to choose	3.35	3.72 ^a^	3.21 ^b^	3.27 ^b^	3.22 ^b^	<0.0001
I compare labels to choose products with the highest nutritional value	3.28	3.65 ^a^	3.15 ^cb^	3.05 ^c^	3.22 ^b^	<0.0001
I purchase more expensive pasta, because I think that the price goes along with the quality	3.36	3.9 ^a^	3.19 ^b^	3.25 ^b^	3.12 ^b^	<0.0001
In order to improve the health-promoting benefits, fiber can be added to the pasta	3.34	3.69 ^a^	3.31 ^b^	3.38 ^b^	3.08 ^c^	<0.0001
The taste of pasta is more important to me than its health-promoting benefits	3.21	3.30 ^ab^	3.14 ^bc^	3.35 ^a^	3.09 ^c^	0.004
It is vital to consume enough pasta	2.97	2.97 ^ab^	2.86 ^b^	2.87 ^b^	3.09 ^a^	0.004

Means with the same letter are not significantly different; ANOVA post-hoc Waller-Duncan K-ratio t Test.

**Table 6:** Profile of segments in terms of statements referring to information on the pasta label.

Statements	Mean	Quality-Oriented 1	Sensory-Oriented 2	Convenience-Oriented 3	Neutral 4	*p*-Value
price	4.29	4.71 ^a^	4.42 ^c^	4.53 ^b^	3.73 ^d^	<0.0001
shelf life	4.27	4.74 ^a^	4.46 ^b^	4.54 ^b^	3.61 ^c^	<0.0001
product name	4.08	4.67 ^a^	4.16 ^b^	4.12 ^b^	3.56 ^c^	<0.0001
weight	3.96	4.59 ^a^	3.75 ^c^	4.11 ^b^	3.53 ^d^	<0.0001
cooking time	3.91	4.53 ^a^	3.52 ^c^	4.37 ^b^	3.40 ^c^	<0.0001
product composition	3.85	4.66 ^a^	3.64 ^b^	3.63 ^b^	3.51 ^b^	<0.0001
producer	3.84	4.56 ^a^	3.66 ^b^	3.79 ^b^	3.45 ^c^	<0.0001
information on health effects	3.77	4.61 ^a^	3.50 ^c^	3.71 ^b^	3.36 ^c^	<0.0001
calorific value	3.72	4.56 ^a^	3.47 ^b^	3.48 ^b^	3.41 ^b^	<0.0001
information on the fiber content	3.63	4.46 ^a^	3.38 ^b^	3.37 ^b^	3.32 ^b^	<0.0001
quality label	3.61	4.55 ^a^	3.41 ^b^	3.24 ^c^	3.29 ^bc^	<0.0001
recipes for pasta dishes	3.39	4.13 ^a^	2.56 ^d^	3.63 ^b^	3.22 ^c^	<0.0001

Means with the same letter are not significantly different; ANOVA post-hoc Waller-Duncan K-ratio t Test.

**Table 7:** Profile of the segments in terms of statements referring to cereal products with added fiber.

Statements	Mean	Quality-Oriented 1	Sensory-Oriented 2	Convenience-Oriented 3	Neutral 4	*p*-Value
They facilitate a healthy lifestyle	3.70	4.09 ^a^	3.65 ^b^	3.77 ^b^	3.37 ^c^	<0.0001
May lower the negative consequences of an inadequate diet	3.61	4.04 ^a^	3.63 ^b^	3.73 ^b^	3.18 ^c^	<0.0001
I can prevent disease by consuming such products regularly	3.52	4.02 ^a^	3.37 ^c^	3.66 ^b^	3.16 ^d^	<0.0001
There is a need to add fiber to cereal products	3.52	3.94 ^a^	3.48 ^b^	3.54 ^b^	3.20 ^c^	<0.0001
The addition of fiber to bread and pasta raises their calorific value	2.96	3.34 ^a^	2.99 ^b^	3.09 ^b^	2.34 ^c^	<0.0001
The addition of fiber to cereal products worsens their taste	2.95	3.12 ^a^	2.58 ^c^	2.94 ^b^	3.06 ^ab^	<0.0001

Means with the same letter are not significantly different; ANOVA post-hoc Waller-Duncan K-ratio t Test.

**Table 8:** Profile of segments in terms of statements referring to pasta with added fiber compared to the same pasta but without fiber.

Statements	Mean	Quality-Oriented 1	Sensory-Oriented 2	Convenience-Oriented 3	Neutral 4	*p*-Value
Is more expensive	3.68	4.07 ^a^	3.48 ^b^	4.08 ^a^	3.25 ^c^	<0.0001
Is healthier	3.61	4.09 ^a^	3.59 ^c^	3.78 ^b^	3.14 ^d^	<0.0001
Has a higher nutrient content	3.52	3.99 ^a^	3.36 ^c^	3.64 ^b^	3.20 ^d^	<0.0001
Is less calorific	3.50	3.96 ^a^	3.51 ^b^	3.57 ^b^	3.10 ^c^	<0.0001
Is more difficult to find in shops	3.42	3.71 ^a^	3.38 ^b^	3.51 ^b^	3.18 ^c^	<0.0001
Has a better taste	3.31	3.95 ^a^	2.76 ^c^	3.31 ^b^	3.21 ^b^	<0.0001
Looks worse	2.94	3.18 ^a^	2.54 ^c^	2.89 ^b^	3.04 ^ab^	<0.0001
Has a more visually attractive packaging	2.94	2.86	2.96	3.06	2.91	0.45

Means with the same letter are not significantly different; ANOVA post-hoc Waller-Duncan K-ratio t Test.

## Data Availability

Not applicable.
